# Remarks on the Pathology and Treatment of Fever

**Published:** 1847-02

**Authors:** James C. Harris

**Affiliations:** Wetumpka, Alabama


ART. IV.—*Remarks on the Pathology and Treatment of Fever.* By JAMES C. HARRIS, M.D., of Wetumpka, Alabama.

In entering upon the discussion af this part of our subject, we shall confine our remarks to those varieties of the disease commonly designated intermittent, remittent, and yellow fever, as embracing all the forms which during the summer and fall seasons prevail in South Alabama. With the exception of the latter variety, which seems to require for its production a higher and longer continued temperature, and a nearer approximation of the dew point to the same, all these fevers appear to be the result alone of malaria, aided, perhaps, by atmospheric vicissitudes and constitutional predisposition.

That the above positions are, as a general rule, correct, is evident from their prevalence at the same seasons, and in the same localities, and from their frequently running into one another. Upon this subject Professor Caldwell, speaking of the yellow fever of Philadelphia in 1803, observes: "As it receded from the low ground and malignant atmosphere of water street, it became more and more mild and manageable, until its evanescent shades in second street were in many instances much lighter than the common remittent of the country." Drs. Rush, Ramsay, and Davidge all speak of the simple intermittent, remittent, and yellow fever as prevailing together, and running into each other, or which was more generally the fact, the former being gradually swallowed up in the latter, establishing the position beyond the possibility of a reasonable doubt, that there is nothing in the atmospheric constitution necessary to the production of intermittents and remittents, that precludes the prevalence of yellow fever. But that any one has hitherto been able to establish an exact identity of cause, we have yet to learn. The mere circumstance that in a great many intermittent and remittent fever districts, yellow fever never appears even sporadically, is of itself sufficient, notwitstanding that remittents may prevail in yellow fever regions, to establish the necessity of some other agent or sensible atmospheric alteration. We know that the power of absorption in the air is increased in proportion to its dryness and temperature, and that vicissitudes in its thermometrical, hygrometrical or barometrical states can seldom occur singly. Thus, for example, when the air is warm and consequently more rarefied, the barometer sinks, whilst its capacity for aqueous vapor is increased. By a careful examination of the meteorological tables kept at New Orleans and Natchez,* during the autumnal months of the years 1839 and 1840, in addition to obtaining a clear comprehension of the foregoing principle, we may learn that notwithstanding the excessive heat and dryness of the summer and fall of the former of those years, the barometrical difference at the same point, and even at different places, was but trifling during all that period; ranging, at New Orleans, in 1830, but little higher than it did at Natchez in 1839, al-

* American Almanac, and Hogg on yellow fever. Western Journal, June, 1840, page 404.

though the mean monthly heat was not much greater at the latter place in 1839, than it was in New Orleans in 1840.

From this it is clear that the geographical position and local peculiarities of Natchez under high and long continued heat and dryness, are such as to supply a sufficiency of moisture by evaporation to produce highly concentrated malaria and by the power of absorption, thus suddenly acquired, approximate the dew point very nearly to the temperature, producing that close, sultry state of the air, and that absence of the morning dews so graphically described by Dr. Monette,* as occurring there during the prevalence of yellow fever in 1839. We are informed by this writer, that at Natchez from the 1st of June, until the last of September of the same year, the thermometer ranged from 85° to 95° in the shade, and at the coolest hour of the night from 75° to 80°. In towns and cities, he continues, the reflected and radiated heat increased the extreme temperature to 88° and 96°, by day, and from 78° to 83° by night, the sea breeze at New Orleans reducing the temperature some two or three degrees lower than it was at Natchez, as was ascertained by comparing the tables of Dr. Tooley, of Natchez, with those kept at the Charity Hospital in New Orleans.

In closing this part of our subject we would remark, that the comparative or total exemption from yellow fever of the Island of Bermuda, the town of Campeachy, the Island of Pettites Coquilles, and the beautiful village of Vidalia, opposite Natchez, as well as of all other cities, towns, and villages within the regions heretofore designated as the common or occasional theatre of yellow fever, seems to us to be dependent upon the absence of malaria in sufficient quantities to co-operate with the atmospheric alterations heretofore indicated. It is alone upon the principle of their joint action that we are able to account for the occasional exemption of the cities of New Orleans, Mobile, and Savannah from yel-

---

* Monette on yellow fever. West. Jour., May, 1842, p. 327.

low fever whilst a brisk trade is maintained between them and the ports of Havana and Vera Cruz, while the disease is raging in the latter cities. So fully is this principle recognized and acted upon, that Commodore Conner, so soon as he is informed of the appearance of yellow fever on board any of the vessels of his command, orders them to be thoroughly cleansed and put to sea; and this is always attended with the happiest results, no more cases occurring after the ships have passed beyond the influence of the malarial land breeze, unless it be an occasional 'light attack,' in the persons of those whose systems had been thoroughly charged with malaria before leaving port.

Respecting the more immediate action of the remote causes of fever, we refer the reader to an article by the writer, on this subject, in this Journal for June, 1846. In this paper one of the positions taken was that the first impression made by malaria upon the system, is upon the blood in the lungs, and through the organic changes and morbid alterations thus effected, weakened action of the heart and perverted nervous action follow as a necessary consequence; that from a law both of the nervous and muscular systems they must act and repose by turns; that, owing to the connection existing between them and all other parts of the system, every paroxysm excites in the system a general disturbance; creating congestions, which end in local inflammation in some one or more of the different organs, and which become a new source of irritation, and aid in perpetuating the circle of morbid phenomena.

In the same communication it was also contended, that many of our most important remedies in fever reached the seat of morbid action through the medium of the circulation. Quinine we suppose to be one of these. Always a 'stimulant,' its peculiar effects upon the skin, the circulation, and the nervous system, depends upon their state at the time of its administration. Upon this subject we are happy to find that we are pretty fully sustained by the recent experiments of Dr. Mendenhall, of Cincinnati, detailed in the American Journal of the Medical Sciences, for July, 1846. This remedy in the hands of those who entertain different views concerning its operation, and who are in the habit of prescribing it at the time of high febrile excitement, to the almost entire exclusion of the lancet, the cold bath, and mercurial cathartics, are, in our opinion, doing more injury to the constitutions of their patients than ever resulted from the employment of any other vegetable remedy. Why, in the treatment of fever, where a furred tongue, nausea, and torpor of the liver and bowels, with yellowness of the skin, exist to a considerable extent, any good results should be expected from the use of a remedy which does not evacuate in any sensible manner, and which, according to some, is a powerful narcotic, is to us passing strange. Quinine has been known to produce symptoms such as to excite in the patient fears of approaching dissolution, while ringing in the ears, giddiness, nausea, deafness, loss of vision, stranguary, and paralysis of the tongue, are occasional effects of its administration. Such a remedy should certainly be prescribed with more caution, and with greater discrimination as respects the different states of the system.

From what has already been advanced it will appear that we do not look upon fever in its incipient stages as having any local organic habitation within the system, becoming local secondarily from violent febrile reactions and the greater weakness of some particular organ. Those most usually affected, according to the report of ten cases of remittent fever, which occurred in the Baltimore Alms House Infirmary, collected and reported by Drs. Anderson and Fricke,* were the brain, the lungs, the heart, stomach, and intestines, spleen, liver, and gall-bladder. Of these, the spleen in every instance gave evidence of enlargement and softening, and in nine out of the ten cases, was of a bluish black color. The liver was also in every instance abnormal, being in color either

* American Journal of the Medical Sciences, for April, 1846.

bronzed, or like that of slate. In seven cases out of the ten in which the gall-bladder was examined, it was found distended with a thick grumous bile resembling molasses, and in the eighth case moderately so, with straw colored bile. Although derangements in other forms of malarial fever, similar to those already described, frequently occur, every system, and every organ in the body becoming occasionally diseased during their progress, death resulting from one or many local inflammations, still, from the want of uniformity in their seat and extent, we are compelled to view them in the light of secondary complications, and treat them accordingly.

*Symptoms.*—Every practitioner is familiar with the symptoms of simple intermittent fever; we shall therefore pass them over, with the single remark, that it must be borne in mind that, in different individuals, the symptoms usually detailed as occurring in regular succession, and at stated periods, frequently prevail in different degrees, and that the several stages in the time they occupy may be in different proportions to one another. But in all, distinct remissions and exacerbations mark the disease. The most frequent varieties in Alabama are the quotidian and the tertian.

An attack of remittent fever is most generally preceded for several days by languor, slight headache, unpleasant taste in the morning, loss of appetite, and pain in the joints.—The first paroxysm is usually ushered in by a slight sensation of coldness in the small of the back, attended with shivering, which sometimes amounts to a light chill, or even an ague. During this stage the tongue is moist and loaded down the centre with a dark brown or light gray fur, and the patient is harassed with intolerable pains in the loins and joints, attended with nausea and vomiting. The pulse is small, soft, and weak, gradually increasing in force and frequency until it rises during the stage of excitement to 130 or 140 in some instances, abating generally during the remission, but never coming down, unless assisted by well directed remedies, to the healthy standard. During this stage the respiration is hurried, the tongue furred in the centre and red around the edges, and dry; excessive irritability of the stomach is also present, the matters ejected by vomiting being generally those most recently swallowed, mixed with bile sometimes of a healthy appearance, at others of a dark or bluish green color. The bowels are variously affected; sometimes they are costive, at others inclined to diarrhœa. When the latter condition obtains, the dejections are frequent, of a reddish cast, containing mucous flocculi which are passed with griping, attended with more or less abdominal tenderness. The urine is scanty and high colored, the thirst great, and the sense of debility extreme, although the patient is able to turn himself in bed, and rise without any assistance. The skin, which is hot and dry, assumes in some cases a jaundiced hue; there is pain in the head, attended with anxiety and restlesness, and, when the fever rises high, delirium.

Where the foregoing symptoms are left to run their course unchecked by any remedial means, low, muttering delirium, subsultus tendinum, picking at the bedclothes, involuntary discharges, coldness of the extremities, and hiccough supervene, and make up the symptoms that close the scene in death .

In this variety, the cold stage is scarcely ever so long or so distinctly marked as it is in intermittent fever, and never returns; there is also an augmentation of all the other symptoms in violence and duration, and although there are slight remissions and exacerabations occurring daily, they are generally, if noticeable at all, most clearly marked every third day.

The inflammatory variety most frequently prevails during a dry hot season, and generally attacks sanguine, plethoric individuals from northern latitudes, and in the most severe and fatal cases is attended with yellowness of the skin and eyes, and vomiting of a brown, blue, or black bile. Its type in either the newly arrived or the native, is much more continued than any other variety. The heat of surface, anxiety, restlessness, and tension of pulse are always greater and more distressing than they are in the ordinary intermittents and remittents of the country.

Ever since we came to this country, during dry, warm spells of weather in the summer and autumn, we have met with cases of the above description, and frequently found the other forms blending with and assuming its type to an extent sufficient to cause us to make some modification in our general plan of treatment.

*Congestive Fever.*—This variety we have long since learned to regard as frequently occurring as a modification in other forms of febrile action, and at any period of the attack; and when complicated with inflammation of the viscera of any of the large cavities it is one of the most severe and fatal of our epidemics. It is observed in places, and at times in which the endemic causes are intense, and concentrated relatively to the state of the predisposition. It is ushered in by a prolonged sense of cold, and universal collapse of the vital powers.* Dr. Parrish† in his strictures upon the term 'congestive,' remarks that Dr. Charles Parry, of Indianapolis, and Dr. R. G. Wharton, of Grand Gulf, have given the most correct account of this variety, as it appears in the localities where they respectively reside. Their account is as follows, and, somewhat modified, suits the character of the disease as it appears throughout the Southern portion of Alabama:

"General coldness of the surface, with a cold clammy perspiration upon the skin of the extremities, intense thirst, with precordial heat, bloody, or red, serous evacuations from the bowels, attended with but little abdominal pain, or tenderness on pressure, great irritability of stomach, with nausea, and frequently vomiting, with a weight and burning at the epigastrium. The respiration is irregular, sighing, and peculiar, and attended with a horrible sense of suffocation, which induces the patient to insist upon getting up, and going into

* Forry on Climate, page 287.
† American Journal of the Medical Sciences, for April, 1845.

the open air; great restlessness with surprising muscular strength throughout, the sufferer being able to rise from bed and walk about the room whilst he is pulseless. The tongue is most usually moist and covered with a dark brown or white fur, and the pulse, when it does exist, is thready and very frequent. The eyes are clear and pearly, and the intellectual faculties are retained with surprising pertinacity. In a large majority of instances, where the attack is ushered in by all or a larger portion of the foregoing symptoms, the powers of the system although unaided by any remedial means, will be found, sooner or later, competent to reaction from the first stage of collapse or chill; but as this form is subject to the general law of periodicity to perhaps as great an extent as any other grade of autumnal fever, twenty-four or forty-eight hours will find the sufferer plunged into another chill or stage of collapse, worse, if possible, than the first, and from which he will be recovered with difficulty."

The reaction, as a general rule, is feeble; the remissions, as remarked by Dr. Boling, "being indicated more by a tendency to the natural temperature, than by any decided mitigation in the other symptoms.*

Having never resided in autumn where yellow fever prevails, we have nothing to offer respecting this formidable scourge of our southern borders, further than what has been already advanced.

*Treatment.*—In this, the first thing to be done is to ascertain if possible what type the fever has assumed or is likely to take on, and until this is accomplished any plan of treatment must be of a doubtful, if not of a positively injurious tendency. As remittents may by judicious treatment be converted into intermittents, these, on the other hand, for the want of a correct understanding of their character, may assume the remittent, or even the more formidable congestive type.

* American Journal for April, 1846, page 308.

Quinine seems of late to constitute no inconsiderable portion of the therapeutic means of many of our southern brethren. Unaided it can, in our opinion, be relied upon in intermittent fever only during the first two or three paroxysms, and not even then provided there be hepatic derangement, which is almost universally present at the commencement of most of our cases. Complications, too, of a local character early arise, requiring other means of cure.

Purgatives and the lancet will now have to be conjoined with quinine—the latter to be used according to the judgment of the practitioner, derived from the amount of local pain and other signs of inflammation. In a large majority of cases, in the first instance, quinine to arrest periodicity, and calomel and the lancet to break up local inflammations, will be found sufficient to give relief. But as this disease is peculiarly liable to return from slight causes, any irregularity in diet, or change in the weather being found sufficient to bring on a relapse, and as they sometimes under such circumstances induce at length a depraved state of the fluids, owing to which they continue to recur, a different plan of treatment becomes necessary. Quinine will no longer answer the purpose.

The blood requires to be renewed, and for this purpose *iron*, or some of its preparations, will be found preferable, associated with some vegetable bitter. The carbonate in pills, and the tincture of the muriate are the preparations which we have most generally used. In some very obstinate cases, and where a hebdomadal tendency was clear, we have effected some very remarkable cures by correcting the secretions with an occasional mercurial cathartic or two, anticipating the chill with quinine, and then, during the remission, administering some one of the preparations of iron. But where all these means fail, which is sometimes the case, and where dropsy or phthisis is threatened, we would advise a change of residence, and the adoption of such means as are best calculated to restore the deranged viscera to healthy action, and impart tone and vigor to the system.

We might here very properly make some remarks upon the treatment of congestive fever—*algid intermittent;* but as we have reserved this variety for a more particular consideration hereafter, we proceed to notice the other varieties. In these, the use of the lancet and mercurial cathartics, as a general rule, are much more imperiously demanded than in the preceding. The former we employ for the purpose of reducing arterial action, warding off local determinations, and relieving them when in existence. The latter we administer for the purpose of disgorging the liver and removing local congestions; and notwithstanding the odium which has been heaped upon *calomel*, we cannot but regard it as one of the most efficacious remedies in the treatment of fever. That it has been frequently abused we do not doubt; but this ought not to be urged against its judicious employment. So long as practitioners continue its administration in three and four grain doses, in combination with opium or some one of its preparations, hour after hour, and day after day, until mercurialization results, it must continue to be unpopular. It is, however, believed that better views are beginning to prevail with the profession, and that the fear of hypercatharsis and watery purging from ten grain or scruple doses of calomel uncombined, is fast giving way. That such doses are much more certain to produce free, consistent bilious purging, with much less danger of permanent injury to the system, we are thoroughly satisfied. We would then commence the cure of either remittent or inflammatory fever, where the pulse was full and tense, and the other attendant symptoms seemed to require it, with a free bloodletting, and the administration of twenty or thirty grains of calomel. The latter we direct to be carried off, if necessary, in the course of ten or twelve hours, by a dose of castor oil. After the operation of the first dose, the calomel should be repeated daily, or every other day, according to the urgency of the symptoms, the condition of the tongue and the effects produced. During this time, should slight pain or tenderness arise in the abdomen, it may be very successfully combated by the application of warm mush or pepper poultices. Should the bowels incline to watery purging, ten grains of calomel in combination with a grain of the acetate of morphia, and the same dose repeated at the end of two or three hours, will generally be found to answer the ends desired. We make it a general rule, during the fall season, to check the operation of calomel with a dose or two of morphia, where there is the least disposition shown to watery evacuations. This course we consider indispensably necessary to the preservation of the strength of our patient. When the tone of the system is sufficiently lowered, and the secretions are established, which in remittent fever, in most cases, will take place in three or four days, quinine, given in ten or fifteen grain doses, will be likely to result in a complete solution of the fever. Although we agree that there is no absolute or fixed standard of symptoms necessary to constitute a remission, still in our experience it has only been in cases where the remissions have been clear and well marked, and where an evident tendency to a solution existed, that we have derived any decided benefits from the use of this remedy.

In the inflammatory variety, so far as our observation extends, quinine is but seldom if ever admissible. The type here is evidently 'continued,' requiring during the early stage, the liberal use of the lancet and mercurial cathartics, conjoined with the cold bath, affusion or sponging. For the intolerable pain in the head, cups between the shoulders, or to the temples, and cold applications, will be found to give considerable relief. We have also in some instances derived considerable advantage from the warm bath, which has reduced the pulse, softened the skin, and relieved the general restlessness more effectually than cold baths.

*Congestive Fever.*—In this type of fever, quinine, the cold dash, and mustard poultices are the leading remedies. As regards the use of quinine, our practice has been to give it in anticipation of the chill, rather than during its actual existence. We commence eight or ten hours before the expected cold stage, no matter whether it occurs daily or every other day, and give from five to six grains every hour or every two hours until twenty-five or thirty grains have been taken. This is generally attended with good effects. Sometimes in combination with each dose we give from the eighth to the fourth of a grain of the acetate of morphia, and from five to six grains of capsicum, and direct, at the same time, a strong tea of this article, which, in our hands, relieves the nausea to a greater extent than any other that we have ever tried.

The use of the *cold dash*, we are informed, originated in this State with Drs. Fearn and Erskine, of Huntsville, and may be safely practised after the following manner: The patient being first stripped and placed upon the floor, cold water is then poured upon him from a pitcher, or thrown with some force from a bucket all over the body. He is then wiped dry, placed in bed and well covered; from ten to fifteen grains of quinine are then administered.—Should warmth and natural perspiration not succeed in the course of one or two hours, the dash and the quinine are repeated and seldom fail to restore action. When there is hepatic derangement, or local inflammation, calomel in combination with quinine becomes necessary; and calomel, quinine and morphia are demanded where there is either a laxity of the bowels, or a disposition to serous evacuations.

The 'cold dash,' so far as we are informed, has been but little used by the physicians of Southern Alabama; but that it is a safe and valuable remedy, and entirely applicable in the first or second paroxysm of an algid intermittent, or in the stage of collapse early supervening in any variety of fever, we are thoroughly convinced. We would earnestly recommend it as well calculated to bring about reaction under such circumstances.

During the stage of oppression or collapse, the external application of heat by hot stones, or bottles filled with hot water, together with frictions to the extremities and surface with dry mustard, and with capsicum in spirits, sinapisms and blisters to the spine and extremities, aided by the internal administration of the most powerful stimulants, have all been practised time immemorial. Dr. Baldwin, of this State, has recommended the application of mustard poultices in this condition of fever, and speaks in strong terms of their efficacy. (See this Journal for January, 1845.) Quinine and calomel are also administered in suitable doses, during and after their application, and the practice is represented as being attended with very good results.

Emetics during the fall season, owing to the irritability of the stomach and the disposition to collapse, we consider as positively contraindicated. Piperine is also considered objectionable on the ground that it is apt to irritate the mucous membrane of the stomach, and thereby add to the nausea and vomiting so often attendant upon fevers.

Of the other measures called for in the treatment of fever, we shall only remark that blisters, ice, cold water, and Dover's powder, are all remedies of great value in their proper place.

In conclusion, we would remark that the success of practitioners in the treatment of fever, will be in proportion to their judgment, experience, and power of adapting their remedies to the different stages of the disease. No physician possessing these qualities will adhere blindly to any particular plan, but will modify his measures so as to meet the exigencies of each case. It is not possible for any man long to pursue the practice of physic without having forcibly impressed upon him, that every new epidemic is a new problem, which he must solve. The plan of treatment pursued so successfully last year is found ineffectual this; and the one which, after many trials, has at length been adopted in the disease of the present season, may be unsuited to the disease which another season shall bring with it. The conclusion is, that the physician who is true to the high interests of his profession must continue to be a student to the end of his days, carefully noting the varying phases of disease, and so shaping his remedies as to suit their altered characters. The foregoing remarks on autumnal fevers are submitted with a deep sense of their imperfection, but in the hope that something of practical utility may be found in them, and that they may afford aid at least to the young practitioner in his responsible duties at the bed-side.

November, 1846.

